# Development of neoplasms in pediatric patients with rheumatic disease exposed to anti-tumor necrosis factor therapies: a single Centre retrospective study

**DOI:** 10.1186/s12969-018-0233-1

**Published:** 2018-03-14

**Authors:** Alexandra Okihiro, Rachana Hasija, Lillia Fung, Bonnie Cameron, Brian M. Feldman, Ronald Laxer, Rayfel Schneider, Earl Silverman, Lynn Spiegel, Rae S. M. Yeung, Shirley M. L. Tse

**Affiliations:** 1 University of Alberta, Edmonton, Canada; 2 Harrison Pediatric Rheumatology & Autoimmunity Clinic, Mumbai, India; 3 William Osler Health System, Brampton, Canada; 4 Division of Rheumatology, SickKids, University of Toronto, 555 University Ave, Room 8253 Burton Wing, Toronto, ON M5G 1X8 Canada

**Keywords:** Anti-TNF, Juvenile idiopathic arthritis, Uveitis, Polyarteritis nodosa, Malignancy

## Abstract

**Background:**

Anti-TNF (Tumor necrosis factor) therapy is effective in treating pediatric patients with refractory rheumatic disease. There is however a concern that anti-TNF usage may increase the risk of malignancy. Reports on specific types of malignancy in this patient population have been emerging over the past decade, but there is a need for additional malignancy reports, as these events are rare. Therefore, a retrospective chart review was performed on the biologic database of pediatric rheumatology patients at The Hospital for Sick Children (SickKids) from 1997 to 2013 for neoplasms, patient demographic information and rheumatologic treatment course.

**Findings:**

6/357 (1.68%) rheumatology patients treated with anti-TNF therapy between 1997 and 2013 developed neoplasms. One patient had two malignancies. One patient had a benign neoplasm. Cases were exposed to etanercept, infliximab or both. Neoplasms developed late after anti-TNF exposure (median 5.0 years) and infliximab treatment was associated with a shorter time to malignancy. The neoplasms identified were as follows: 2 renal clear cell carcinoma, 1 pilomatricoma, 1 nasopharyngeal carcinoma, 1 Ewing’s sarcoma, 1 hepatic T-cell lymphoma, 1 lymphoproliferative disease.

**Conclusions:**

The malignancy rate at our centre is low, however more than half of the neoplasms identified were rare and unusual in the pediatric population. The 5-year malignancy-free probability for patients with juvenile idiopathic arthritis (JIA) treated with biologic therapy was 97% from our database. Long-term screening for rare neoplasms is important as part of the safety monitoring for any pediatric rheumatology patient receiving anti-TNF therapy.

## Introduction

The benefit of anti-tumor necrosis factor (TNF) in refractory rheumatic disease is well established [1, 2]. However, there remains a concern for long-term safety since the United States Food and Drug Administration (FDA) reported an increased risk of malignancies in children exposed to anti-TNFs [3]. There have been extensive studies done which suggest that there is no overall increased in malignancy rate in these patients treated with biologics [4–6]. Instead, it has been suggested that the neoplasms occurring in this group have a preponderance towards hematological malignancies [7, 8] and more recently, data on children developing specific solid organ tumors is emerging [9]. As the incidence of neoplasms in rheumatic disease patients exposed to anti-TNFs is limited, characterization of neoplasms in this subset of patients is important to guide malignancy screening recommendations in anti-TNF treatment follow up care. In this study, we describe six children with rheumatic disease who developed one or more neoplasms during or following exposure to anti-TNF treatment at our centre. Additionally, a subset of patients with JIA from the biologic registry were analysed for the probability of neoplasm development after biologic treatment.

## Findings

### Materials and methods

A retrospective review was performed on the Rheumatology Biologic Registry at SickKids. Patients’ medical information, treatments and imaging were retrieved from clinical charts and the registry. The study was approved by the SickKids Ethics Review Board. A nonparametric statistical Kaplan Meier event curve was created to display the probability of neoplasms in patients with JIA exposed to anti-TNFs (confidence interval 95%).

## Results

A search of our registry found 357 pediatric patients with a diagnosed rheumatic disease who were exposed to biologics between January 1997 and August 2013. Biologic exposure included etanercept (52%), infliximab (31%), adalimumab (16%), golimumab (2%), certolizumab (1%), anakinra (11%), canakinumab (4%), tocilizumab (5%), abatacept (4%) or rituximab (3%) with 21% of the patients exposed to more than one biologic. 295 of these patients had a diagnosis of JIA including: 93 RF (rheumatoid factor)-negative polyarthritis, 63 systemic JIA, 39 enthesitis related arthritis, 30 extended oligoarthritis, 29 RF-positive polyarthritis, 18 psoriatic arthritis, 14 persistent oligoarthritis, and 9 undifferentiated. The remaining patients had the following diagnoses: 23 uveitis, 10 vasculitis, 4 chronic recurrent multifocal osteomyelitis, 4 juvenile dermatomyositis, 4 systemic lupus erythematosus, 4 inflammatory bowel disease associated arthritis, 3 autoinflammatory disease/periodic fever, 3 sarcoidosis and 7 other. Six patients with one or more neoplasms during or after anti-TNF exposure were identified resulting in a malignancy rate of 1.68%. Patient demographics, medications and neoplasm characteristics are summarized in Table 1. As a group, the cohort was exposed to etanercept (*n* = 1), infliximab (*n* = 3) or both (*n* = 2). The median time to neoplasm from anti-TNF initiation was 5.0 years. Patients D and F were actively receiving biologic therapy right up until diagnosis of neoplasm. As a group, the median time to neoplasm from anti-TNF cessation was 4.4 years. All 6 patients received methotrexate. The median time to neoplasm from methotrexate initiation was 5.4 years. Patient D did not receive any other concomitant medication. Patient E and F received cytotoxic chemotherapies (etoposide, cyclophosphamide, azathioprine) and two patients Patient B and E received other non-anti-TNF biologic therapies (rituximab, anakinra). The neoplasms identified were 2 renal clear cell carcinoma, 1 pilomatricoma, 1 nasopharyngeal carcinoma, 1 Ewing’s sarcoma, 1 hepatic T-cell lymphoma, 1 lymphoproliferative disease. In the JIA patients, the neoplasm rate was 1.35% with a neoplasm reporting rate of 3.68 events per 1000 patient-years.

**Table 1:** Summary of pediatric rheumatology patient demographics, neoplasms and medications

Patient ID/ gender	Age at diagnosis of rheumatologic disease (years)	Age at diagnosis of neoplasm (years)	Neoplasm	Anti-TNFα (dose/Duration)	Time from Rheumatologic diagnosis to neoplasm (years)	Time from start of anti-TNFα to neoplasm (years)	Concomitant and previous DMARD, cytotoxic agent and other Biologic (years)	Other concomitant and previous medications	Family history of malignancy
A/Female	Extended oligo JIA (1.6 years)	17	Ewing’s sarcoma	etanercept 25 mg biweekly × 2.6 years	16.7	5.3	methotrexate (7.2), leflunomide (0.2)	naprosyn, prednisone, folic acid	Unknown
B/Female	Poly JIA (8.2 years)	24	renal clear cell carcinoma	etanercept 25 mg weekly × 4.8 years, infliximab 400 mg x one dose	15.8	10.6 from etanercept, 4.4 from infliximab	rituximab^a^ (2 doses), methotrexate (6.2), leflunomide (0.2), sulfasalazine (0.9), azathioprine (1.2), cyclosporine A (1.5)	naprosyn, prednisone, hydroxychloroquine, amitriptyline, oxycodone, depo-provera, folic acid	Unknown
C/Female	Extended oligo JIA with anterior uveitis (2.0 years)	14	benign pilo-matricoma	infliximab 200–500 mg monthly × 0.25 years	12.7	1.3	cyclosporine A (3.2), azathioprine (4.3), methotrexate (6.0), leflunomide (2.8)	hydroxychloroquine, acetazolamide, alendronate, iron, calcium	Multiple family members with colon cancer
D/Male	Idiopathic uveitis (10.8 years)	16	naso-pharyngeal cancer	infliximab 300–400 mg monthly × 3.3 years	5.3	3.3	methotrexate (4.7)	folic acid	Maternal grandfather with lung cancer; Maternal grandmother with cervical cancer
E/Female	Systemic JIA (4.7years)	16	hepatic T-cell lymphoma	etanercept 10 mg biweekly × 0.1 years, infliximab (10 mg/kg) monthly × 1.7 years	11.8	9.9 from etanercept, 8.6 from infliximab	anakinra^a^ (1.9), cyclosporine A (0.9), methotrexate (3.6), tacrolimus (5.0), thalidomide (0.6), etoposide (6 doses)	prednisone	Unknown
F/Male	Polyarteritis Nodosa (1.1 years)	14/19	lympho-proliferative disease (age 14); renal clear cell carcinoma (age 19)	infliximab (100 mg) monthly × 4.8 years	14.1 to lympho-proliferative disease; 18.6 to renal clear cell carcinoma	4.8 to lympho-proliferative disease; 9.3 to renal clear cell carcinoma	methotrexate (1.2), azathioprine (unknown), cyclophosphamide (1.5)	prednisone, enalopril, amlodipine, sulfamethoxazole, ranitidine, testosterone, protropin, nutropin, insulin, metformin, ferrous fumarate	Mother with breast cancer (deceased at age 31); Half-brother with suspected autoimmune lympho-proliferative disorder
Median (IQR)	3.4 (1.7–7.3)				16.3 (15.3–17.9)	5.0 (3.6–7.7)	methotrexate: 5.4 (3.9–6.1)		

*JIA* = juvenile idiopathic arthritis

^a^Other Biologic (non-anti-TNF)

### Case descriptions

**Fig. 1:**
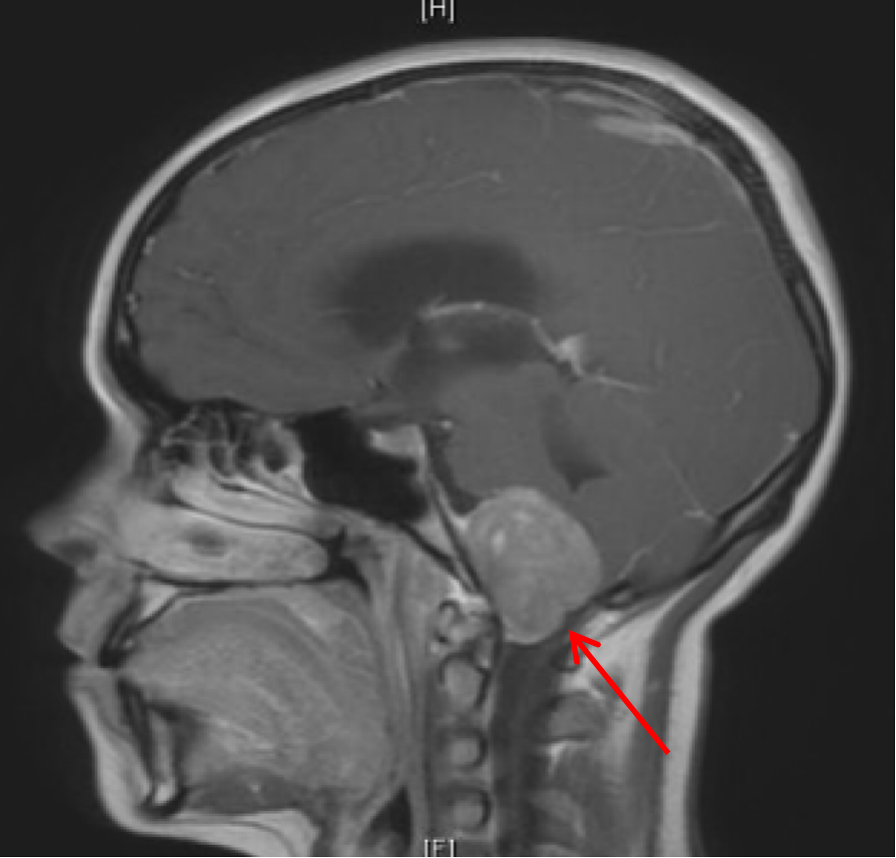
MRI head of a 17-year-old female who developed a basal skull Ewing sarcoma 5.3 years after starting etanercept therapy

Patient A is a 17-year-old female with extended oligoarticular JIA who presented with symptoms of dysphagia, hoarseness, headaches, vomiting and significant weight loss. Physical examination demonstrated left-sided facial weakness, papilledema, ataxic gait, and cerebellar signs localized to the left. Imaging and biopsy investigations revealed a small, vimentin positive, round blue cell tumour suggestive of Ewing’s sarcoma compressing the brainstem (Fig. 1). The patient had previously received etanercept for her JIA. The patient is now deceased.

Patient B is a 24-year-old female with RF-negative polyarticular JIA who presented with progressive vomiting and diarrhea. Abdominal ultrasound revealed a renal mass. Renal biopsy was positive for renal clear cell carcinoma (Fuhrman grade 2). The patient had previously received etanercept and a single dose of infliximab for her JIA.

Patient C is a 14-year-old female patient with persistent oligo JIA and antinuclear antibody (ANA)-positive anterior uveitis presented with a 1 cm erythematous lesion later diagnosed as a pilomatricoma. The patient had previously received infliximab for her JIA and uveitis.

**Fig. 2:**
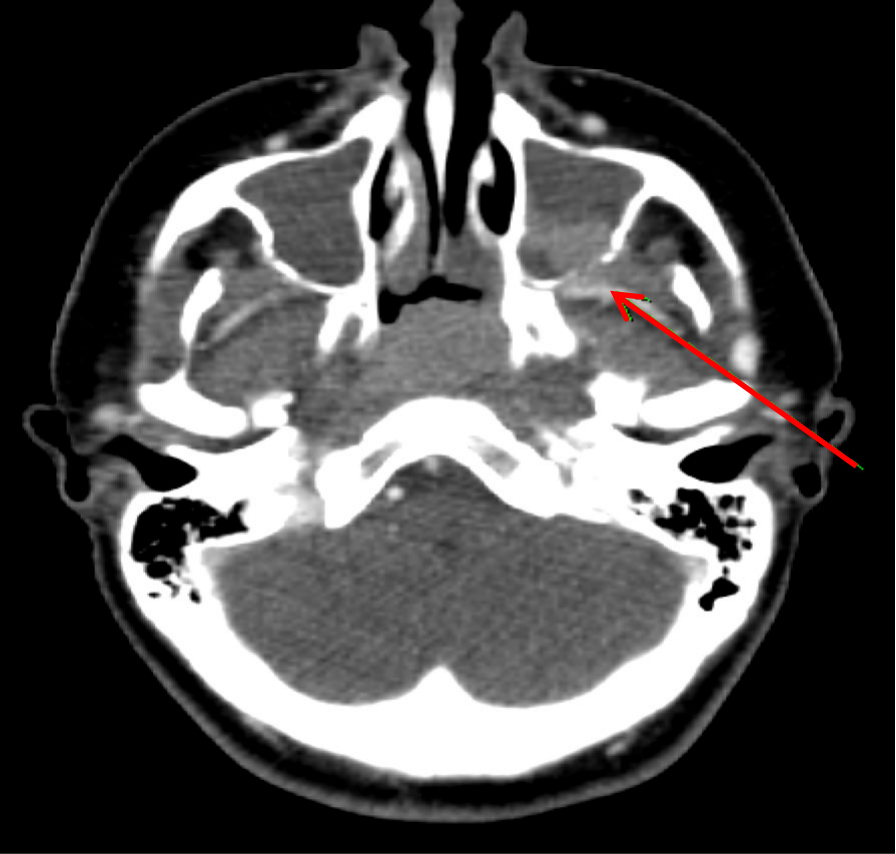
Head CT with contrast of a 16-year-old male who developed a nasopharyngeal carcinoma 3.3 years after starting infliximab infusions

Patient D is a 16-year-old male patient with idiopathic uveitis who presented with complaints of left-sided neck pain and noisy breathing. Nasal polyp and adenoid biopsy revealed a diffuse nasopharyngeal carcinoma (Epstein Barr virus positive) (Fig. 2). There was localized bony destruction and cervical lymph node involvement with no evidence of distal spread. The patient was Caucasian and denied tobacco or alcohol use. The patient had received infliximab up until diagnosis of the malignancy.

Patient E is a 16-year-old female patient with systemic arthritis and recurrent macrophage activation syndrome (MAS) who presented with fever, left upper quadrant pain, splenomegaly and thrombocytopenia. She was hypotensive and tachycardic on admission. Bone marrow aspirate and biopsy revealed hepatosplenic T-cell lymphoma. The patient had previously received etanercept and Infliximab. She died of complications shortly after a bone marrow transplant.

Patient F is a 14-year-old male patient with systemic polyarteritis nodosa (PAN) who underwent a tonsillectomy due to obstructive sleep apnea. Biopsy was consistent with post-transplant lymphoproliferative disease (PTLD), even though the patient had not undergone a transplant. At age 19, echogenic renal lesions were discovered on routine imaging. He was diagnosed with left renal cell carcinoma. He had previously received infliximab up until his first malignancy diagnosis. The patient denied any tobacco or alcohol use. Of note, the patient’s half-brother was being followed for suspected autoimmune lymphoproliferative syndrome (ALPS).

## Discussion

We report six pediatric rheumatology patients with neoplasms following anti-TNF exposure initiated during a period of more than 15 years. The neoplasms at our centre developed late after drug exposure with a median of 5.0 years from anti-TNF onset. Specifically, the patients who developed renal clear cell carcinoma and hepatic T-cell lymphoma had their malignancies develop remote from anti-TNF usage. The time to malignancy was shorter with infliximab although etanercept was trialed first in our patients receiving multiple anti-TNF treatment. One North American study involving six pediatric centres (ours included) examined the time of neoplasm in JIA patients after anti-TNF therapy [10]. Five malignancies (hepatosplenic T-cell lymphoma, polymorphic post-transplantation lymphoproliferative disease, renal cell adenocarcinoma, Ewing’s sarcoma, endometrial adenocarcinoma) were reported with the mean time to malignancy after anti-TNF exposure was 1.49 (median 0.5) years, which is shorter than our findings. These patients were also exposed to many other concomitant medications including other biologics and methotrexate. We also report a neoplasm rate in our JIA patients to be 3.68 events per 1000 patient-years. For comparison, another Canadian group summarized the findings of seven studies and reported a neoplasm risk of 1.3 events per 1000 patient-years in JIA patients exposed to anti-TNFs [11].

Most of the neoplasms at our centre were unusual for the pediatric population. Benign pilomatricomas (Patient C) are quite uncommon, representing 0.12% of integument system neoplasms [12]. Nasopharyngeal carcinomas (Patient D), particularly those with EBV positive pathology, are most common in adult patients from Asian countries. When they occur in North American children, it is an extremely rare event with a reported incidence of 0.5 million person-years for patients aged 0-19 years [13]. Renal clear cell carcinomas (Patient B and F) are extremely rare in pediatrics with an overall age-adjusted incidence in the general population of 0.01/100,000 [14]. The pathology differs from adult renal cell carcinoma which is typically seen in the sixth decade of life and is associated with tobacco use. Interestingly, the FDA’s Adverse Event Reporting System (AERS) identified another pediatric patient with renal cell carcinoma following anti-TNF treatment [3]. To the best of our knowledge, this is the first report of benign pilomatricoma and nasopharyngeal carcinoma in a pediatric patient following anti-TNF exposure for rheumatic disease management.

**Fig. 3:**
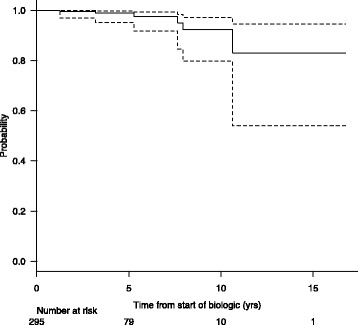
Kaplan-Meier event curve depicting probability of neoplasm development from start of biologic therapy in patients with JIA (*n* = 295) from the Rheumatology Biologic Registry. Dashed lines represents 95% Confidence Interval

As previously mentioned, patients who developed neoplasms from our study as well as in the literature had received multiple concomitant medications other than anti-TNFs, making establishment of causality difficult. All of our study patients were exposed to methotrexate and all but one patient (Patient D) received other concomitant medications, including other biologics in some patients. It has been speculated that methotrexate as an immunosuppressant may increase the rate of malignancy in patients with JIA, although this has yet to be proven [15]. Two of our patients received additional treatment with etoposide, cyclophosphamide and azathioprine, which have known cytotoxigenicity. Additionally, our patient who developed lymphoproliferative disorder had a second-degree relative with suspected ALPS and therefore may have had a genetic predisposition to malignancy. Also, the underlying disease process in rheumatic disease may further complicate our ability to determine any relationship in between anti-TNF and malignancy risk. It has been suggested that there may be a background incidence of malignancy in children with inflammatory disease activity, irrespective of biologic treatment. In patients with JIA, there are studies that report an approximate 2 to 4-fold increase in malignancy risk [16–19] as well as those that do not endorse this relationship [10, 20]. We are unable to comment on the overall risk of malignancy in patients with rheumatic disease, as we do not have a comparator cohort from our centre. As previously mentioned, numerous studies in patients with JIA have suggested that anti-TNF therapy does not increase the risk of neoplasm development [4–6]. Additional large-scale studies concerning infliximab therapy and pediatric inflammatory bowel disease have also shown no association with increased malignancy risk [21]. A report summarizing over 180,000 adult patients with rheumatoid arthritis treated with anti-TNFs also concluded that there is no increased cancer risk [22]. We examined patients diagnosed with JIA and the probability of developing a neoplasm at certain time periods after the start of biologic therapy (Fig. 3). For example, the survival curve demonstrated a 97% probability (Confidence Interval 92–97) of being neoplasm free 5.3 years after biologic exposure for patients of this subset, further supporting that malignancy development in this population is rare.

We acknowledge that our study is limited by including patients with different underlying diagnosis with varying exposures to multiple anti-TNF agents, other biologics and concurrent medications. Also, we are unable to make conclusions about our centre’s pediatric rheumatology patients who were not exposed to biologic therapy, as we did not perform this control. Our study is further limited by the fact the both pediatric rheumatic disease and malignancies in childhood are rare events in themselves and as such, larger populations would be necessary to make any true conclusions about a causal relationship. Additionally, the Kaplan-Meier curve depicting time from biologic start to neoplasm in patients with JIA may be limited due to fewer patient numbers at longer follow up times.

In summary, we report six pediatric rheumatology patients who developed neoplasms during or after treatment with anti-TNF agents. Four of the patients had rare neoplasms for the pediatric population. The neoplasms occurred late after anti-TNF initiation, and infliximab was associated with a shorter time to malignancy compared to etanercept. Within the JIA subset, patients had an extremely favourable probability of being neoplasm free 5 years after initiation of biologics. It still remains unclear whether there is any causal relationship between anti-TNF agents and malignancy risk, as there are many potential contributing factors including the potential effect of disease activity and the contribution of concomitant medications. Additional long-term studies with larger populations should be completed to identify the types of malignancies that occur in this population. Appropriate malignancy screening at each clinical visit, including an appropriate history and physical exam especially for constitutional symptoms, masses, lymphadenopathy and new skin symptoms should continue as part of anti-TNF monitoring.

## Abbreviations

ALPS: Autoimmune lymphoproliferative syndrome
ANA: Antinuclear antibody
JIA: Juvenile idiopathic arthritis
MAS: Macrophage activation syndrome
PAN: Polyarteritis nodosa
PTLD: Post-transplant lymphoproliferative disease
RF: Rheumatoid factor
TNF: Tumor necrosis factor
